# Exercise participation has increased in patients with Rheumatoid Arthritis: A cross-sectional comparison between two Dutch RA cohorts

**DOI:** 10.31138/mjr.29.4.199

**Published:** 2018-12-18

**Authors:** Julia M. Weijers, Sanne A.A. Rongen-van Dartel, Piet L.C.M. van Riel

**Affiliations:** 1 Radboud University Medical Centre, Radboud Institute for Health Sciences, IQ Healthcare, Nijmegen, The Netherlands,; 2 Department of Rheumatology, Bernhoven Hospital, Uden, The Netherlands

**Keywords:** Exercise participation, physical activity, recommendation, rheumatoid arthritis

## Abstract

**Objective:**

This study evaluates exercise participation in patients with rheumatoid arthritis (RA) and the percentage of patients that meet the recommended level of physical activity (at least 150 minutes per week moderate-intensity physical activity) in two cross-sectional questionnaires in 2013 and 2016 in two Dutch RA cohorts.

**Methods:**

In 2013, a cross-sectional study was performed among 740 patients with RA from seven outpatient clinics from the Dutch DREAM registry. Subsequently in 2016, 498 patients with RA of the outpatient clinic of the Bernhoven Hospital (member of the DREAM registry) participated in a similar study. In both years, patients filled in an identical questionnaire about exercise participation (frequency and duration). In 2016, items about self-efficacy to become more physically active were added to the questionnaire.

**Results:**

In 2016, patients with RA spent significantly more minutes per week in exercise activities compared to 2013: 180 (150–450) and 120 (60–225) minutes per week, respectively (P<0.001). The percentage of patients with RA who met the recommended physical activity level increased from 25% in 2013 to 57% in 2016. Almost half (44%) of the non-exercisers reported feeling confident to become more physically active.

**Conclusion:**

Compared to 2013, RA patients participated in 2016 more frequently and spent more minutes per week in exercise activities. This resulted in a higher percentage of patients who met the recommended physical activity level. A personalized physical activity program, with a focus on identifying barriers and setting personal goals, might further increase the physical activity level of patients with RA.

## INTRODUCTION

Participation in physical activities is important for everybody, as it has many beneficial effects on physical as well as mental health outcomes.^1–5^ The American College of Sports Medicine and the American Heart Association recommend all healthy adults to participate in moderate-intensity aerobic physical activity for a minimum of 30 minutes on at least five days a week.^6,7^ Physical activity is defined as any bodily movement resulting in energy expenditure,^8^ including exercise as well as non-exercise activities. Exercise is a subset of physical activity that is planned, structured, and repetitive, and has as objective the improvement or maintenance of physical fitness.^8^ A large part of the general population is not physically active enough, according to the international recommendation for physical activity.^9–12^

Similar to the general population, patients with a chronic disease are also insufficiently physically active, including patients with rheumatoid arthritis (RA).^13^ It has been shown in several studies that patients with RA are similar or even less physically active compared to healthy controls.^14–16^ For patients with RA, physical activity is especially important, as joint inflammation and pain could both lead to functional impairments, which could result in a less physically active lifestyle. Physical inactivity in turn may lead to further progression of the disease.^17^ In addition to the overall health benefits, physical activity in patients with RA has an effect on reduction of the elevated cardiovascular risk, increasing muscle strength and physical functioning and reduction in disability, pain and fatigue.^18–26^

In recent years, attention and awareness of being physically active has increased. In 2010, the European League Against Rheumatism (EULAR) recommendation for cardiovascular disease risk management (CVRM) in patients with RA did not discuss diet or physical activity, but these topics were only mentioned in the research agenda.^27^ The 2015 American College of Rheumatology (ACR) guideline for the Treatment of RA does also not (yet) address lifestyle-related (i.e., physical therapy) aspects, due to resource limitations.^28^ Research on the role of exercise in the management of chronic diseases, including RA, has increased considerably in the past decade, including the development of exercise programs or interventions to increase physical activity.^29–34^ In recent years, at the ACR and EULAR annual conferences, (lack of) physical activity was more frequently an addressed topic, which reflects the increased research interests. As a result, in 2016, the EULAR adopted lifestyle recommendations, including regular exercise, in their updated CVRM recommendation.^35^

Besides the development of interventions to improve physical activity levels and exercise participation in general and the adoption of lifestyle advice in treatment recommendations, national and international authorities supported the promotion of physical activity in society. In 2013, the European Council adopted the European Network for the Promotion of Health-Enhancing Physical Activity (HEPA Europe). This network aims to strengthen and support efforts and actions that increase physical activity and improve the conditions favourable to a healthy lifestyle.^36–38^ As an example, the Dutch government launched the National Prevention Program (NPP) in 2014; a joint effort by six ministries, municipalities, businesses and civil society organizations. The program consists of laws and regulations and various health programs financed by the government, aimed to reduce the prevalence of chronic diseases by focusing on obesity and physical activity.^39^ Also, within Dutch hospitals and at general practices, many initiatives have taken place to increase exercise participation among patients with chronic diseases. Despite increased attention for being physically active, it is unknown whether patients with RA actually became more physically active in daily practice in the past years.

Therefore, this study will estimate whether exercise participation - and as a consequence, the percentage of patients with RA that meet the recommended level of physical activity - has changed in recent years. Second, this study aims to gain insight into self-efficacy of patients with RA of the outpatient clinic who do not meet the recommended level of physical activity to become more physically active.

## MATERIALS AND METHODS

### Design

Two cross-sectional studies were performed at two points in time, each among a Dutch cohort of patients with RA. Both cohorts were part of the Dutch RhEumatoid Arthritis Monitoring (DREAM) registry in the Netherlands. The DREAM registry is a multicentre prospective ongoing cohort study of RA patients started in 2003 (http://www.dreamregistry.nl/en).

### Assessment of exercise participation in 2013

Between April 2013 and October 2014, 3800 patients with RA from seven outpatient clinics from the DREAM registry were invited to fill in an online questionnaire about their physical activity level. Patients had been invited via 1) the website of the Arthritis foundation in the Netherlands and via a patient magazine and a newsletter especially for people with rheumatic diseases, or 2) via their online electronic patient database called Rheumatology Online Monitor Application (ROMA).

The questionnaire consisted of questions about patient characteristics (age, gender, and disease duration) and physical activity. Physical activity was measured using the Short QUestionnaire to ASsess Health enhancing physical activity (SQUASH). The SQUASH is a reliable and validated Dutch questionnaire to measure physical activity for one week.^40^ For this study, we only focused on exercise participation, which is one aspect of physical activity besides household activities, commuting or activities at school or work. Therefore, we only included data regarding exercise activities in the analyses. The respondents were asked to refer to an average week in the last month. Total minutes of exercise were calculated for each exercise activity by multiplying frequency (days per week) by duration (minutes per day). Weekly total amount of exercise was calculated by taking the sum of the total minutes per week for each exercise activity.

### Assessment of exercise participation in 2016

In December 2016, all 726 patients with RA of the out-patient clinic of Bernhoven, a hospital in the South of the Netherlands and member of the DREAM registry, received a questionnaire on paper about their exercise participation. After two months, in February 2017, a reminder was sent to all patients who did not respond to the questionnaire.

This questionnaire consisted of identical questions about exercise participation as in 2013 completed with questions about patients’ characteristics, exercise participation and self-efficacy to become more physically active. The purpose of the questionnaire was to screen patients with RA for their current exercise participation and self-efficacy to become more physically active. Patients who do not meet the recommended level of physical activity and who have self-efficacy to increase this level, will be invited to participate in a personalized exercise program. The patients were not aware that they were identified for inclusion in the exercise program.

### Self-efficacy to become physically active

The questionnaire of 2016 consisted of the following five questions about self-efficacy to improve or sustain the current level of physical activity: “I am able to increase my physical activity level”; “I feel able to accept the challenge to increase my physical activity level”; “I am convinced to be physically able to perform at least 5 days a week for 30 minutes moderate physical activities”; “How much confidence do you have that you will become or sustain physically active under supervision for the next 3 months?” and “How much confidence do you have, that you will sustain an increased physical activity level after exercise under supervision?” All questions had four response options: from *totally not agree* to *fully agree* or from *no confidence* to *much confidence*. To investigate to what extent patients with RA perceived their disease as a barrier to exercise, the following question was included in the questionnaire of 2016: “Are you hindered by your rheumatic disease during exercise?” This question had five response options: from *not at all* to *a great extent*.

### Statistical analysis

Differences in patient characteristics between 2013 and 2016 were analysed using an independent student t-test (age), a Mann-Whitney U test (disease duration) and a Chi-square test (gender). Differences in weekly minutes of exercise between both cohorts were tested using a Mann-Whitney U test, and changes is proportion of patients who met the recommended level of physical activity was analysed using a Chi-square test. Pearson correlation coefficients were calculated to assess relationships between patient characteristics and exercise participation. To correct for potential confounding, a sensitivity analysis (Cochran-Mantel-Haenzel test) was performed for the variables age, gender and disease duration. Analyses were performed with SPSS, version 25. Results are presented as median plus interquartile ranges unless stated otherwise.

## RESULTS

### Inclusion of respondents

In 2013, 740 of 3800 patients with RA (20%) filled out the online questionnaire. In 2016, 48% (352 of 726) of the patients with RA of the outpatient clinic in Bernhoven initially responded to the questionnaire. After sending a reminder, this percentage increased to 69% (498 of 726).

### Patient characteristics

Patient characteristics of the included patients with RA in 2013 and 2016 are shown in *Table 1*. On average, patients with RA who participated in 2016 were 8 years older compared to patients included in 2013, 64 ± 13 versus 56 ±12 years respectively (P<0.001). The proportion of women was ten percent higher in 2016 compared to 2013: 65% versus 55% respectively (P=0.018). Disease duration was shorter in 2016 compared to 2013, 8 (3–13) years (median (IQR)) in 2016 and 8 (4–9) years in 2013 (P<0.001). Correlations of age, gender and disease duration with the amount of exercise participation were very small and the inequity of the distributions of these variables between the 2 cohorts is small. Therefore, it can be concluded that weekly amount of exercise was not confounded by patient characteristics.

**Table 1.** Patient characteristics, exercise duration and number of exercise activities

	**2013**	**2016**
**Patient characteristics**	n=740	n=498
Age, years, mean ± SD	56 ± 12	64 ± 13 ^**^
Female, number (%)	409 (55)	325 (65)^*^
Disease duration, years, median (p25–p75)	8 (4–19)	8 (3–13) ^**^
**Exercise duration, number (%)**	n=740	n=498
No exercise participant, performed 0 minutes per week exercise	302 (41)	127 (26) ^**^
Exercise participant, performed at least > 0 minutes per week exercise	438 (59)	371 (74) ^**^
Performed < 150 minutes per week exercise	256 (35)	86 (17) ^**^
Performed ≥ 150 minutes per week exercise	182 (25)	285 (57) ^**^
Minutes per week, median (p25–p75)	120 (60–225)	180 (150–450) ^**^
**Exercise activities, number (%)**	n=438	n=371
Participated in one type of exercise	259 (59)	152 (41)^**^
Participated in two types of exercise	137 (31)	141 (38)^*^
Participated in three types of exercise	42 (10)	78 (21) ^**^

* between group difference P<0.05,

** between group difference P<0.001

### Exercise participation

Based on the time of exercise participation, patients with RA could be divided in three groups: *did not participate in exercise* (zero minutes per week); *participated in exercise, but less than the recommended physical activity level of 150 minutes per week*, and *participated 150 or more minutes per week in exercise*.

Patients with RA who participated in exercise in 2016 (n=371), spent significantly more minutes per week exercising compared to the exercise participants in 2013 (n=438), 180 (150–450) and 120 (60–225) minutes per week respectively (P<0.001). The percentage of patients with RA who participated in exercise for a minimum of 150 minutes per week (the recommended level of physical activity) increased from 25% (182/740) in 2013 to 57% (285/498) in 2016 (P<0.001).

**Figure 1.**
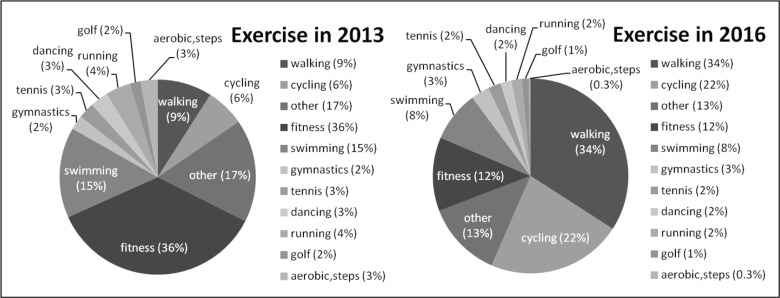
Overview of types of exercise in which patients with RA participated in 2013 and 2016.

The percentage of patients with RA who participated in an exercise activity (at least > 0 minutes per week) increased from 59% in 2013 to 74% in 2016. In 2013, 59% (259/438) participated in one type of exercise, 31% participated in two different types of exercise and 10% participated in three different types of exercise. More patients in 2016 participated in more than one type of exercise. The percentage of patients who participated in two or three different types of exercise increased to 38% and 21% respectively (*Table 1*). Fitness, swimming, cycling and walking were the most popular exercise activities among patients with RA. The popularity of each activity changed between 2013 and 2016 (*Figure 1*).

### Self-efficacy to become physically active

**Figure 2.**
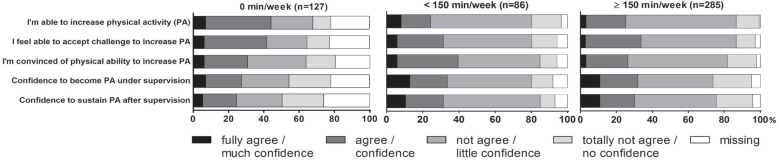
Self-efficacy to increase physical activity level among three different levels of exercise participation.

In 2016, five questions about self-efficacy to improve or sustain the current level of physical activity were adopted in the developed questionnaire. No remarkable differences in self-efficacy to increase physical activity level existed between the three groups (zero minutes/week, <150 minutes/week or ≥150 minutes/week of exercise participation) (Figure 2). However, of the 127 patients with RA who did not participate in exercise, more than one fifth (20–26%) did not fill out the questions about self-efficacy. The percentage missing decreased to 6% among the patients who exercised less than 150 min/week, and decreased even further to 3% among the patients who participated in exercise for more than 150 minutes/week. The question whether RA is a barrier to participate in exercise was more frequently unanswered in patients who did not participate in exercise, compared to patients who did participate in exercise, 39% versus 5% respectively. Of the other 61% (77/127) who answered the barrier-related question, 75% (58/77) confirmed that RA is a barrier to participate in exercise. Patients who participated in exercise and reported their rheumatic disease less frequently as a barrier to perform exercise activities amounted to 56% (197/352).

Of the patients who did not participate in exercise, 44% (56/127) filled out that they felt able to increase their level of physical activity and accept the challenge to do this. A quarter had the confidence to become physically active with guided supervision, and a quarter also had the confidence to remain physically active after guided supervision. In general, the patients with RA who had already met the recommended level of physical activity had less self-efficacy to increase their physical activity level further.

## DISCUSSION

According to the results of this study, patients with RA participated in 2016 more frequently and spent more minutes per week in exercise activities compared to 2013. Fifty-nine percent of the patients with RA exercised in 2013, which increased to 74% in 2016, with fitness, swimming, cycling and walking as the most popular exercise activities in both years. The percentage of patients with RA that met the recommended level of physical activity, based on exercise participation, increased from 25% in 2013 to 57% in 2016.

It is difficult to compare the percentages of patients who meet the recommended level of physical activity reported in previous studies with the percentages found in this study. Data about physical activity were gathered in different ways; objectively or subjectively. Even when we compare our results with other studies that measured physical activity subjectively, different questionnaires were used, or other definitions or calculations of physical activity were applied. The majority of these studies reported physical activity in general, while this study focused on exercise participation, which is one element of physical activity. Previous studies which subjectively measured physical activity levels of patients with a rheumatic disease reported a range between 14% and 40% of patients who met the recommend level.^15,41,42^

Because exercise participation was investigated twice in different patient populations of the DREAM registry, using the same questions to assess exercise participation and similar statistical analyses, it is more easily to make a comparison between two points in time in this study (2013 and 2016). Additionally, we measured exercise participation in a population that was not involved in a specific intervention to improve their physical activity level (such as an exercise program). Therefore, we measured the change in exercise participation over time instead of change caused by an exercise intervention.

We are aware of the differences in age, gender and diseases duration of the respondents between 2013 and 2016. The lower age of patients in 2013 could be explained by the fact that we used an online questionnaire: only patients with sufficient digital skills were able to fill out the online questionnaire in 2013, which were, in general, younger patients. In 2013, the questionnaire was distributed online and in 2016 patients could fill in the questionnaire on paper. This change in distribution method could have caused the increase in response rate between 2013 and 2016. Even before sending a reminder to the patients who had not responded to the questionnaire on paper in 2016, the response rate was already 48%. Although there was a high response rate, our results are only based on the patients who responded to the questionnaire, and response bias could have occurred. It is plausible that a part of the patients who did not respond to the questionnaire in 2013 or in 2016 did not participate in exercise, did not have any interest in physical activities, or filling in questionnaires in general. This phenomenon was also discussed in previous studies from Van Loon et al. and Drivsholm et al. who both reported a higher level of physical inactivity in non-responders on a questionnaire within two large cohorts.^43,44^ This is supported by our observation that those patients who answered that they did not participate in exercise did not fill out the questions about self-efficacy and about RA as barrier to participate in exercise as frequently as those who did participate in exercise. Therefore, we think that the percentage of all patients with RA which are sufficiently physically active will probably be lower.

Increased attention and awareness of the benefits of being physically active in the society, at outpatient clinics and among general practitioners, and the adoption of lifestyle advice in the EULAR recommendations may have contributed to the increase in the percentage of RA patients who met the recommend level of physical activity between 2013 and 2016.^27,35^ The rheumatology outpatient clinic in Bernhoven organized meetings for patients about lifestyle (including physical activity, smoking and diet); a network of physiotherapists in the region of the hospital supporting patients to be physically active and several patient organization groups (part of the Dutch Arthritis Foundation) in the region organized weekly exercise activities for their members.

The large increase in patients with RA who met the recommended level of physical activity (>100%, while the increase in minutes per week was just 50%) could also be partly explained by the fact that some patients in 2013 were just below the 150-minute threshold. An explanation for the differences in the percentages of certain types of exercise between 2013 and 2016 could be that, in 2013, patients could choose their exercise activities from a list of 59 different types of exercise. In 2016, only the top 10 most frequently chosen types of exercise from 2013 were shown, and one option of “other”. We reduced the response options of type of exercise from 59 options to 10 options, since the other options (no. 11–59) were hardly chosen in 2013.

Still, a quarter of the patients with RA reported in 2016 that they did not participate in exercise. An explanation could be that patients with RA, or patients with a rheumatic disease in general, need to be assisted more to overcome barriers to implement exercise and other physical activities in their daily life. 75% of the patients with RA who did not participate in exercise confirmed that RA is a barrier for exercise activities. Besides disease related barriers, (fear of) pain and fatigue, lack of knowledge and uncertainty about safe exercises and injury prevention are also mentioned as barriers for being physically active.^45^ Education and advice from healthcare professionals (i.e., a physiotherapist or rheumatologist [nurse]) could help to identify and overcome barriers in those who do not meet the recommended level of physical activity.

Almost half of the patients with RA of the outpatient clinic in this study who did not participate in exercise felt able to become more physically active. However, only a quarter of the inactive patients with RA reported they had the confidence to become more physically active in the upcoming three months. A more personalized approach, based on coaching and shared decision-making to set personal physical activity goals, could increase compliance and reduce barriers and improve confidence to being physically active. Such a personalized approach would be in line with the recently published EULAR recommendations for physical activity promotion and delivery in the management of people with inflammatory arthritis and osteoarthritis.^46^ A new study should examine whether a personalized physical activity program is effective integrate physical activities, including exercise participation, in daily life of patients with a rheumatic disease. In conclusion, patients with RA participated in 2016 more frequently and spent more minutes per week in exercise activities compared to 2013. Despite more attention and awareness in recommendations and the promotion of physical activity in society, a large part of the patients with RA is still insufficiently physically active. A more personalized approach to identifying barriers and setting personal physical activity goals could be effective to increase and sustain the level of physical activity in patients with a rheumatic disease.
